# Jmjd6, a JmjC Dioxygenase with Many Interaction Partners and Pleiotropic Functions

**DOI:** 10.3389/fgene.2017.00032

**Published:** 2017-03-16

**Authors:** Janice Kwok, Marie O’Shea, David A. Hume, Andreas Lengeling

**Affiliations:** The Roslin Institute and Royal (Dick) School of Veterinary Studies, University of EdinburghEdinburgh, UK

**Keywords:** JmjC domain, demethylation, hydroxylation, chromatin, PSR, splicing, transcription, pausing

## Abstract

Lysyl hydroxylation and arginyl demethylation are post-translational events that are important for many cellular processes. The jumonji domain containing protein 6 (JMJD6) has been reported to catalyze both lysyl hydroxylation and arginyl demethylation on diverse protein substrates. It also interacts directly with RNA. This review summarizes knowledge of JMJD6 functions that have emerged in the last 15 years and considers how a single Jumonji C (JmjC) domain-containing enzyme can target so many different substrates. New links and synergies between the three main proposed functions of Jmjd6 in histone demethylation, promoter proximal pause release of polymerase II and RNA splicing are discussed. The physiological context of the described molecular functions is considered and recently described novel roles for JMJD6 in cancer and immune biology are reviewed. The increased knowledge of JMJD6 functions has wider implications for our general understanding of the JmjC protein family of which JMJD6 is a member.

## Introduction

The jumonji domain-containing protein 6 (Jmjd6) is a member of the large family of JmjC domain-containing metalloenzymes. These proteins are ferrous iron (Fe^2+^)- and 2-oxoglutarate (2OG)-dependent dioxygenases that can catalyze hydroxylation and demethylation reactions on protein and nucleic acid substrates (Hausinger, 2004; Loenarz and Schofield, 2008; Markolovic et al., 2015; Martinez and Hausinger, 2015). The JmjC protein subfamily of histone demethylases perform essential functions as regulators of transcriptional control, chromatin structure, epigenetic inheritance, and genome integrity (Klose and Zhang, 2007; Pedersen and Helin, 2010; Kooistra and Helin, 2012; Fedeles et al., 2015). Jmjd6 is one of the smallest JmjC proteins (Fadok et al., 2000; Cikala et al., 2004; Tibrewal et al., 2007; Hahn et al., 2010) and appears to interact with multiple protein substrates in distinct molecular pathways. Jmjd6 contributes to the regulation of histone demethylation, transcriptional polymerase II promoter pause release and mRNA splicing (Chang et al., 2007; Webby et al., 2009; Liu et al., 2013) through its ability to catalyze two types of reactions, lysyl hydroxylation and *N*-methyl argininyl demethylation. The latter catalytic mechanism is controversial but of particular importance because until recently Jmjd6 was proposed to be the only enzyme capable of catalyzing arginine demethylation. Arginine methylation is quantitatively one of the most extensive protein methylation reactions in mammalian cells (Bedford and Clarke, 2009). Analysis of the human arginine methylome identified 8030 high-confidence monomethylarginine sites on 3300 proteins in human embryonic kidney (HEK) 293 cells (Larsen et al., 2016). Arginine monomethylation is only the prerequisite for the enzymatic catalysis of arginine dimethylation, so the vast number of differently arginine-methylated proteins in cells suggests that this post-translational modification (PTM) is as widespread as phosphorylation and ubiquitination (Clarke, 2013; Biggar and Li, 2015; Poulard et al., 2016; Blanc and Richard, 2017). Since arginine methylation is a reversible and dynamic PTM, an understanding of the underlying enzymatic writer, reader and eraser systems for protein methylation has become a key priority area of investigation (Bedford and Clarke, 2009; Carlson and Gozani, 2014; Sylvestersen et al., 2014). The potential role of Jmjd6 in erasing arginine methylation is therefore topical, if controversial (Mantri et al., 2010; Böttger et al., 2015).

This review summarizes our current knowledge about Jmjd6 interaction partners, its identified catalytic substrates and proposed molecular functions. We will discuss how a single JmjC protein can target so many different substrates. We will highlight studies from the past 15 years which have significantly contributed to our current understanding of Jmjd6 functions and identify new links and connections between the three main proposed functions of Jmjd6 in transcriptional control that have, as yet, not received much attention. This will cover the cross-talk and synergy between histone demethylation, promoter-proximal pause release of polymerase II and RNA splicing. Finally, we will discuss Jmjd6 functions in a physiological context and review its recently described novel roles in cancer and immune biology.

## The Jmjd6 Protein – Basic Structural Features and Potential Functions

Jmjd6 was discovered 17 years ago and named phosphatidylserine receptor (PSR, Ptdsr) because it appeared to function as a transmembrane receptor for recognition and clearance of apoptotic cells (Fadok et al., 2000; Segawa and Nagata, 2015). Subsequent studies in mice, Hydra and cell lines demonstrated that the ‘PSR’ function was incorrectly assigned and that Jmjd6 is predominantly located in the cell nucleus (Böse et al., 2004; Cikala et al., 2004; Cui et al., 2004; Mitchell et al., 2006; Wolf et al., 2007). By cloning the homologous gene in Hydra, Cikala et al. (2004) recognized that the protein sequence contains a central jumonji C (JmjC) fold, and predicted that the protein would function as Fe^2+^ and 2OG-dependent dioxygenase. Subsequent confirmation of this activity (Chang et al., 2007; Webby et al., 2009), led to the renaming of the protein as Jmjd6.

**FIGURE 1:**
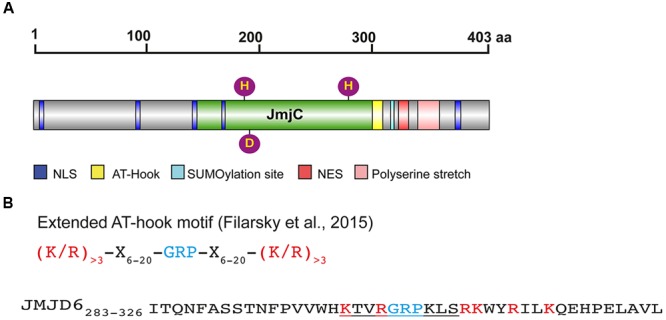
**Protein domains and motifs of Jmjd6. (A)** Schematic presentation of the full-length JMJD6 protein (403 amino acids, UniProt ID Q6NYC1) with central JmjC domain (green), nuclear localization sites (NLS, blue), nuclear export signal (NES, red), predicted SUMOylation site (light blue), and C-terminal poly serine (pink). Fe^2+^ complexing residues are shown as purple balls (His187, Asp189, His273). Scale indicates the length of the 403 amino acid (aa) comprising protein **(B)** Sequence of an extended AT-hook-like motif in JMJD6. Shown on the top line is the consensus sequence of an extended AT hook (eAT) after (Filarsky et al., 2015). GRP indicates the glutamine-arginine-proline tripeptide core motif which is surrounded in close proximity by at least three basic leucine (L) and/or arginine (R) residues. X indicates any amino acid in a distance of 6–20 amino acids from the GRP core motif. Bottom line: peptide eAT-hook-like sequence of JMJD6 (residues 283–326). GRP core motif is depicted in blue, basic K and R amino acids in red. The central/canonical AT-hook sequence is underlined. Note that the JMJD6 eAT-hook sequence with basic amino acids (K/R) is only extended distally to the GRP core motif sequence. Thus, Jmjd6 appears to have a hybrid AT hook sequence motif as discussed in the text (see The Jmjd6 Protein – Basic Structural Features and Potential Functions).

Jmjd6 is conserved from mammals to yeast (Hahn et al., 2008). The central JmjC domain (residues 141 to 305, in human JMJD6, UniProt ID Q6NYC1, **Figure 1A**) forms the typical cupin or double-stranded β-helix fold (DSBH) shared by all known 2OG-dependent dioxygenases (Clissold and Ponting, 2001; Loenarz and Schofield, 2008; Hong et al., 2010; Mantri et al., 2010). The DSBH fold forms a barrel-like structure with eight anti-parallel β-strands forming the major and minor β-sheets in which the Fe^2+^ binding site of the catalytic center is located at the open end of the barrel. The residues forming this Fe^2+^ binding site (His187, Asp189, and His273) follow the conserved HXD/E(X_n_)H motif characteristic of JmjC family members and are essential for the enzymatic activity of Jmjd6 as demonstrated by mutagenesis analyses (Chang et al., 2007; Webby et al., 2009; Liu et al., 2013; Heim et al., 2014). Two protein crystal structures have been reported for Jmjd6 (Hong et al., 2010; Mantri et al., 2011). The first structure was obtained from a truncated Jmjd6 protein lacking the C-terminal located poly serine rich region (polyS, residues 340–365 in Q6NYC1) which was reported to be incompatible with crystal formation (Mantri et al., 2010). Interestingly, this structure shows Jmjd6 crystallized as a homo-dimer and reveals details about the interfacing β-strands and α-helixes that are involved in dimer formation. Two α-helixes positioned at the N- and C-terminal ends of the protein (residues 61–68 and 322–334 in human JMJD6) are essential for Jmjd6 homo-dimerization (Mantri et al., 2010; Wolf et al., 2013). Homo-oligomerization of JmjC domain-containing proteins has also been reported for other family members. For example, the factor inhibiting HIF (FIH), which is structurally similar to Jmjd6, spontaneously forms dimers in solution. Other examples include the tRNA hydroxylase, TYW5, and the ribosomal oxygenase 1 (RIOX1, also known as NO66), which can oligomerise to form dimers or tetramers, respectively (Kato et al., 2011; Tao et al., 2013). Jmjd6 can also form trimers, tetramers, and pentamers by homo-multimerization (Hahn et al., 2010; Han et al., 2012; Wolf et al., 2013). The formation of these multimers has been proposed to require the catalytic activity of Jmjd6 (Han et al., 2012). Like other JmjC hydroxylases, the protein is capable of carrying out autohydroxylations (Mantri et al., 2012) which may be necessary for the intermolecular cross-linking of Jmjd6 monomers (Han et al., 2012). As a consequence these multimers are partially resistant to sodium dodecyl sulfate treatment and are visible as discrete protein fragments on western blots (Tibrewal et al., 2007; Hahn et al., 2010; Wolf et al., 2013). Importantly, the expression of these multimers varies in different cell types, which may confuse analysis of the specificity of Jmjd6 antibodies in protein immunoprecipitation or western blot analysis. Since the discovery of Jmjd6 the whole field has been misled by cross-reactivity of anti-Jmjd6 antibodies (Fadok et al., 2000; Böse et al., 2004; Wolf et al., 2007; Böttger et al., 2015). Many commercially available anti-Jmjd6 antibodies recognize unknown fragments above the 50 kD monomer fragment in *Jmjd6* knockout cells (Hahn et al., 2010). Therefore, new anti-Jmjd6 antibodies must be validated using *Jmjd6*-deficient cells. The cellular function of the different Jmjd6 multimers is unknown. They may be formed under different physiological conditions in different nuclear compartments. Electron microscopy studies performed with recombinant Jmjd6 protein have suggested that monomers polymerize into ring-like structures (Wolf et al., 2013). The formation of these structures *in vivo* and the question of whether they are part of larger protein scaffolds or complexes requires further investigation.

Besides its catalytic JmjC domain, Jmjd6 has a poly-serine (polyS) stretch domain at its C-terminus (residues 340–365 in Q6NYC1, **Figure 1A**). This domain is missing in alternatively spliced variants of Jmjd6 which are likely to have distinct biological functions (Hahn et al., 2008; Wolf et al., 2013). The polyS domain is important for the subnuclear localization of Jmjd6 (Wolf et al., 2013); in its absence Jmjd6 variants are predominantly localized in the fibrillar center of the nucleolus. In immunoprecipitation experiments, Jmjd6 without the polyS domain interacts with nucleolar proteins (Wolf et al., 2013), suggesting a function for the domain in nuclear/nucleolar shuttling of the protein. We have observed different amounts of nucleolar and nucleoplasmatic Jmjd6 in individual cells in culture (Hahn et al., 2010). Whether these different Jmjd6 nuclear expression patterns are dependent on the cell cycle or on other physiological stimuli needs to be determined.

Jmjd6 has five predicted nuclear localization sites (NLSs) and one nuclear export signal (NES) (**Figure 1A**). Two of the NLS overlap the JmjC domain and may not be topologically accessible *in vivo* (Cikala et al., 2004; Cui et al., 2004). Three NLS have been validated using Jmjd6 reporter deletion constructs (Cikala et al., 2004). Other sequence motif predictions in Jmjd6 which still demand experimental validation are a putative sumoylation site (K317 in Q6NYC1, Hahn et al., 2010) and an AT-hook domain (residues 299–317 in Q6NYC1, Cikala et al., 2004) which has been originally described as a DNA binding motif in the high mobility group AT-hook 1 protein (HMGA1) (Reeves and Nissen, 1990). The AT-hook in HMGA1 facilitates protein binding to the minor groove of DNA (Huth et al., 1997). There is no evidence that Jmjd6 binds to DNA (Hong et al., 2010), but it does interact with RNA. The nuclear staining pattern of Jmjd6 is lost in RNase A treated cells (Hahn et al., 2010) and specific interactions of Jmjd6 with arginine-serine-rich (RS)-domain containing proteins were found to be mediated at least partially through interaction with RNA (Heim et al., 2014). Jmjd6 associates with nascent RNAs in the nucleoplasm, identified as distinct dots by labeling RNA in HeLa cells with 5-fluorouridine (Heim et al., 2014). In the report describing the monomeric crystal structure of Jmjd6, the full-length protein was shown to bind without sequence specificity to random RNA oligos of 21–27 nucleotide length (Hong et al., 2010). Additional evidence of RNA binding to Jmjd6 has been obtained in co-precipitation studies which have analyzed the association of Jmjd6 with mRNA or small nuclear (sn) RNA in relation to its involvement in splicing and transcriptional pausing as discussed below (Boeckel et al., 2011; Liu et al., 2013; Heim et al., 2014). One candidate binding motif identified within Jmjd6 is a variation of the original AT-hook motif (Filarsky et al., 2015). It contains the glycine-arginine-proline (GRP) core motif of the canonical AT-hook but is extended symmetrically in both directions with basic amino acids at a distance of 12–15 residues. The extended AT-hook peptide motif (eAT-hook) was shown to have a higher affinity for RNA than DNA (Filarsky et al., 2015). However, the Jmjd6 AT-hook sequence contains neither a canonical AT-hook nor an eAT-hook motif (**Figure 1B**). The GRP core in Jmjd6 is extended with basic lysine and arginine residues in the C-terminal direction from the core but not in the N-terminal direction as described for eAT-hooks (**Figure 1B**). Thus, Jmjd6 possess a hybrid between a canonical and an extended AT-hook. Whether this peptide motif is directly involved in mediating interactions of Jmjd6 with RNA needs to be investigated using mutagenesis studies.

Many different substrates of Jmjd6 have been reported in the literature, with different levels of evidence (**Supplementary Table S1**). Some studies have provided indirect evidence that Jmjd6 catalyzes demethylation of arginine residues in target proteins using methylation-specific antibodies in immunoprecipitation and/or western blot analyses (**Supplementary Table S1**). Others describe *in vitro* assays with recombinant Jmjd6 protein purified from *Escherichia coli* and peptides derived from candidate protein substrates (Chang et al., 2007; Webby et al., 2009; Webby, 2012; Liu et al., 2013; Unoki et al., 2013; Wang et al., 2014; Gao et al., 2015). To provide definitive evidence for genuine Jmjd6 catalyzed reactions, matrix-assisted laser desorption/ionization (MALDI) mass spectrometry (MS) analyses are required to demonstrate reaction products derived from endogenous proteins. Such *in vivo* evidence has been provided in only three studies. Using liquid chromatography–mass spectrometry/mass spectrometry (LC–MS/MS) analyses Webby et al. (2009) demonstrated that the splicing factor U2 small nuclear ribonucleoprotein auxiliary factor 65-kilodalton subunit (U2AF65) from HeLa cells is lysyl-5-hydroxylated by Jmjd6 at positions K15 and K276. Overexpression of Jmjd6 in HeLa cells resulted in a 5-fold increase in hydroxylation of U2AF65 at K15 thus providing further evidence that U2AF65 is a genuine Jmjd6 substrate *in vivo* (Webby et al., 2009). Similarly, MS analyses indicated that immunoprecipitated Jmjd6 undergoes self-hydroxylation on K167 in HeLa cells (Webby, 2012) and JMJD6 lysyl-hydroxylates endogenous p53 on position K382 in HCT116 colon cancer cells (Wang et al., 2014) (as discussed below).

The evidence that the protein is also catalyzing *N*-methyl-arginine demethylation reactions is less compelling. The proposed arginine demethylation activity of Jmjd6 has been suggested to involve an initial hydroxylation reaction on the *N*-methyl group which yields an unstable hemiaminal intermediate that subsequently fragments into the demethylated arginine residue and formaldehyde as a by-product (Chang et al., 2007; Loenarz and Schofield, 2008; Böttger et al., 2015). Comparison of available structures from JmjC hydroxylases and JmjC histone lysine demethylases (KDMs) with that of Jmjd6 suggests that *N*-methyl-arginine groups might not easily penetrate into the catalytic cavity site of Jmjd6 [for excellent reviews on JmjC protein structure comparisons see (Loenarz and Schofield, 2011; Böttger et al., 2015; Markolovic et al., 2015, 2016)]. Other JmjC proteins can undergo large conformational changes to accommodate different substrates (Scotti et al., 2014). Nevertheless, direct evidence for the proposed *N*-arginine demethylation activity of Jmjd6 remains to be established using endogenous protein substrates and employing either detailed MS-fragmentation studies and/or NMR amino acid analyses.

In conclusion, Jmjd6 is a relatively small JmjC domain containing protein that interacts with RNA and has an enzymatic function as Fe^2+^- and 2OG-dependent dioxygenase. Its nuclear function as lysyl-hydroxylase has been validated on endogenous substrates in different cells but its possible function as an arginine demethylase still awaits experimental validation *in vivo*.

## Expression of Jmjd6 *In Vivo* and Phenotypes Reported in *Jmjd6*-Deficient or *Jmjd6*-Overexpressing Model Organisms

Jmjd6 is expressed during all stages of mouse development. Specific patterns of expression can be detected at embryonic stages E9.5-12.5 in the developing neural tube, somites, heart, gut, limb buds and eyes (Böse et al., 2004; Schneider et al., 2004). In adult mice Jmjd6 is ubiquitously expressed in most tissues at a moderate or low level. More prominent expression can be found in testis, thymus, heart, kidney, liver and skin (Böse et al., 2004). The BioGPS portal which provides public access to the Gene Atlas Affymetrix array sets from 61 mouse and 79 human tissues, organs, and cell lines shows in general a good correlation of *Jmjd6*/*JMJD6* expression between both species (**Supplementary Figure S1**; Wu et al., 2009). *Jmjd6* is expressed throughout the hematopoietic system with significant expression levels in progenitor stem cells of the myeloid and lymphoid lineages. Strong expression levels of *Jmjd6*/*JMJD6* can be found in CD4^+^ and CD8^+^ T cells, B cells, NK cells and monocytes (**Supplementary Figure S1**). This pattern of expression is confirmed in the extensive promoter-based gene expression analysis from the FANTOM Consortium and the RIKEN PMI and CLST (DGT) et al. (2014) where the highest expression of *Jmjd6* was observed in myeloid cells (granulocytes, mast cells, and monocytes).

Loss-of-functions studies in knockout mice have demonstrated that Jmjd6 has essential functions in embryogenesis and tissue differentiation. *Jmjd6* knockout mice die neonatally at the latest (Li et al., 2003; Böse et al., 2004; Kunisaki et al., 2004). In our knockout strain, which was generated on a C57BL/6J genetic background (Böse et al., 2004), we have observed a low rate of embryonic lethality *in utero* (5–6%). *Jmjd6* knockout mice display a range of defects during embryogenesis. From midgestation onward delays in the differentiation of lungs, kidneys, the intestine, the thymus and eyes can be observed (Böse et al., 2004; Kunisaki et al., 2004). Brain development is severely affected with sometimes extensive hyperplasia of brain tissue that leads to exencephaly (Li et al., 2003; Böse et al., 2004). Homozygous knockout mice with severe head and craniofacial malformations are often also anophthalmic with uni- or bilateral absence of eyes (Böse et al., 2004). Erythropoiesis in the fetal liver is blocked at an early erythroblast stage (Böse et al., 2004; Kunisaki et al., 2004). *Jmjd6*^-/-^ mice also show drastic defects in thymocyte differentiation. At embryonic day E18.5 around only 15% of double positive CD4^+^ and CD8^+^ T cells are found in thymus of *Jmjd6*^-/-^ mice in comparison to wild-type littermates, demonstrating early defects in T-lymphopoiesis (Kunisaki et al., 2004). The function of Jmjd6 in thymus development has been analyzed using fetal thymus grafts of wild-type and *Jmjd6*^-/-^ embryos into the renal capsules of athymic nude mice (Yanagihara et al., 2015). The expression of the autoimmune regulator protein, Aire, was ablated in medullary thymic epithelial cells (mTEC) of *Jmjd6* knockout grafts. Aire is a transcriptional regulator protein that induces expression of peripheral tissue self-antigens in the thymus (Peterson et al., 2008), thereby controlling negative selection of self-reactive thymocytes and promoting immunological tolerance. Ablation of *Aire* function prevents deletion of autoreactive T cells and causes multiorgan autoimmune disorders (Chan and Anderson, 2015). Jmjd6 appears to control Aire expression in mTECs by regulating the splicing of *Aire* transcripts (Yanagihara et al., 2015). In the absence of *Jmjd6*, intron 2 of the *Aire* transcript was not effectively spliced out resulting in the generation of a premature stop codon and expression of a truncated and unstable protein. The lack of functional Aire protein expression in *Jmjd6*^-/-^ mTECs generates multi-organ autoimmunity in nude mice that are reconstituted with thymic *Jmjd6* knockout grafts (Yanagihara et al., 2015). Whether the mis-splicing of *Aire* transcripts involves a failure in Jmjd6 mediated hydroxylation of splicing regulatory proteins remains unknown but is one likely molecular explanation of the observed defect (see below).

**FIGURE 2:**
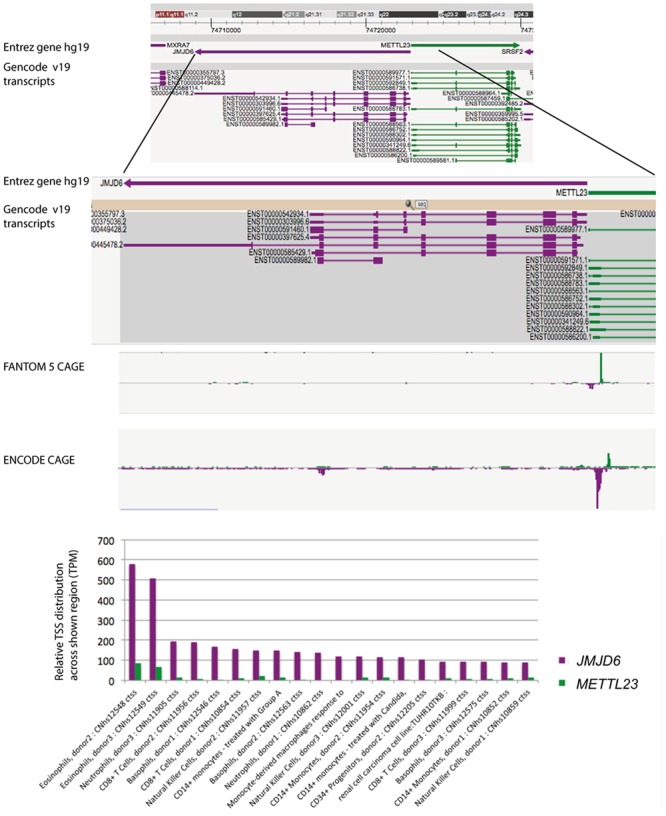
**FANTOM5 analysis of the human locus encompassing the *JMJD6* and *METTL23* loci.** FANTOM5 CAGE sequence data as depicted in the Zenbu browser and described by the (FANTOM Consortium and the RIKEN PMI and CLST (DGT) et al., 2014). Top line: shown are the Entrez hg 19 *JMJD6* (purple arrow) and *METTL23* (green arrow) transcriptional orientations. Sequence positions on human chromosome 17 are indicated on the top. Gencode v19 transcripts for *JMJD6* (purple) and *METTL23* (green) are shown below. Rectangles indicate exons and lines introns. Note: at the proximal and distal borders of the depicted *JMJD6* and *METTL23* loci the Zenbu browser screen view shows parts of the neighboring genes *MXRA7* and *SRSF2* in this gene dense genomic region. FANTOM5 CAGE and ENCODE CAGE sequence peaks are shown in the center. *JMJD6* CAGE sequence peaks are shown in purple, *METTL23* CAGE sequence peaks in green. Bottom graph: relative transcriptional start site (TSS/CAGE) expression data for *JMJD6* (purple) and *METLL23* (green) in blood cells from different donors. Relative TTS tag incidence refers to CAGE sequence tag counts across the locus which are quantified as normalized tags per million (TPM) as previously described (FANTOM Consortium and the RIKEN PMI and CLST (DGT) et al., 2014). CAGE sequence peaks for both genes are partially overlapping in eosinophils, neutrophils, and natural killer cells. The data shown are from the FANTOM5 database publically accessible at the Zenbu browser (http://fantom.gsc.riken.jp/5/) when searching for *JMJD6* or *METTL23*.

Neonatal *Jmjd6* knockout mice die from severe cardiopulmonary malformations involving ventricular septal defects, a double outlet right ventricle, and pulmonary artery hypoplasia (Schneider et al., 2004). Controversy exists over the function of Jmjd6 in apoptotic cell clearance. Two of the reported *Jmjd6* knockout mice are in fact double mutant mice (Li et al., 2003; Kunisaki et al., 2004) which lack, in addition to *Jmjd6*, the neighboring gene *methyltransferase like 23* (*Mettl23*) (Hahn et al., 2008). *Mettl23* encodes a predicted *S*-adenosyl-methionine-dependent methyltransferase and its locus overlaps with *Jmjd6* (Hahn et al., 2008). Both genes are oriented in a head-to-head transcriptional orientation and transcriptional start sites (TSS) for *Jmjd6* and *Mettl23* are in close proximity (**Figure 2**). *Jmjd6* and *Mettl23* cluster in a ‘head-to-head’ transcriptional orientation in all vertebrates from humans to puffer fish (Hahn et al., 2008). Such ancestral conservation of head-to-head gene organization is often correlated with significant overlap of expression patterns and functional associations of the genes involved (Li et al., 2006; Gherman et al., 2009). Although the precise TSS do not overlap, both genes are widely expressed in mouse and human datasets in FANTOM5, and the two genes probably share transcriptional regulatory elements within the CpG island that overlaps the shared TSS region. Nevertheless, in humans their expression is divergent in that *METTL23* is not over-expressed in myeloid cells, and conversely, is high in T cells. Loss-of-function mutations in human *METTL23* have been reported to cause mild non-syndromic or severe syndromic autosomal recessive intellectual disability (ARID). Two truncating mutations in *METTL23* in unrelated Austrian and Pakistan families disrupt the predicted catalytic domain of METTL23 and alter the cellular localization of the protein (Bernkopf et al., 2014). Another loss-of-function mutation in the 5′-region of *METTL23* has been described which is associated with ARID and craniofacial abnormalities in a family of Arabian origin (Reiff et al., 2014). This four base pair deletion affects the coding region of three *METTL23* transcripts and the 5′-UTR of another three alternative transcripts. It is not known whether this 5′ located mutation also effects *JMJD6* expression. The reported craniofacial phenotypes in the Arabian family, which includes a cleft palate, are similar to craniofacial dysmorphologies that are observed in *Jmjd6* knockout mice (Böse et al., 2004; Reiff et al., 2014). The conserved shared regulation of JMJD6 and METTL23 suggests that they might target some substrates in common and could therefore act in shared pathways perhaps as corresponding writer and eraser enzymes. If such is the case, the cell-type specific roles could be impacted by changes in their relative expression.

Essential roles for Jmjd6 in tissue differentiation and embryogenesis have also been demonstrated in zebrafish. A morpholino knockdown of zebrafish *jmjd6* caused severe delays in organogenesis and early lethality with failures to hatch from the egg (Hong et al., 2004). The observed phenotypes correlate with those found in the mouse knockout lines including severe morphological defects in the developing brain, heart, notochord and somites. Increased numbers of apoptotic cells were reported in *jmjd6* morpholino injected embryos in comparison to controls (Hong et al., 2004), but these results do not distinguish whether the underlying cause is an increase in cell death or a failure of clearance.

In *Drosophila*, genetic ablation of dJMJD6 expression produced no obvious phenotypes and homozygous knockout flies are viable and fertile (Krieser et al., 2007). Overexpression of *jmjd6* under ubiquitous promoters resulted in a rotated male genital phenotype similar to fly mutants lacking the developmental apoptosis regulator *head involution defective* (*hid*) (Krieser et al., 2007). Co-expression of *jmjd6* and *hid* in the developing *Drosophila* eye suppressed a small rough eye phenotype that can be induced by overexpression of *hid* alone. These observations led to the hypothesis that dJMJD6 suppresses Hid-dependent apoptosis during the pupal phase of eye development and possibly also in other fly tissues during embryogenesis (Krieser et al., 2007). In *Caenorhabditis elegans* the loss of *jmjd6* has been reported to be associated with mild apoptotic cell engulfment defects (Wang et al., 2003) although this is controversial (Arur et al., 2003). A defect in regenerative axon fusion has been described in *jmjd6* deficient *C. elegans* mutants after laser ablation of mechanosensory neurons (Neumann et al., 2015). However, other worms that lack *jmjd6* have no obvious phenotypes. Together with the genetic studies in *Drosophila* this shows that *jmjd6* is not essential for embryogenesis and development in invertebrates and by inference, the gene has acquired new, non-redundant functions in the vertebrate lineage.

## Biological Functions of Jmjd6 in Transcriptional Control

**FIGURE 3:**
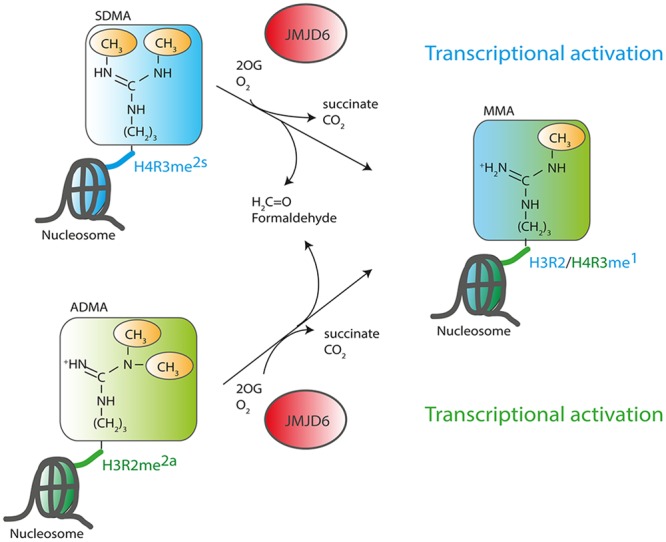
**Function of JMJD6 as a histone arginine demethylase.** JMJD6 has been proposed to demethylate residues at histone H4 arginine 3 (H4R3) and histone H3 arginine 2 (H3R2). Arginine residues can be symmetrically (SDMA) or asymmetrically (ADMA) dimethylated. 2-oxoglutarate- (2OG) and oxygen (O_2_)-dependent demethylation of H4R3^me2s^ and H3R2^me2a^ by JMJD6 produces succinate, CO_2_, formaldehyde and monomethylated arginine residues. Both arginine histone marks, H4R3^me2s^ and H3R2^me2a^ are associated with transcriptionally inactive chromatin. Demethylation by JMJD6 to the monomethyl arginine (MMA) H4R3me^1^ or H3R2me^1^ histone marks is proposed to be associated with transcriptional activation.

The discovery of an enzymatic function of Jmjd6 as 2OG-dependent dioxygenase suggested that the protein plays a role in the epigenetic regulation of chromatin structure and gene expression (Chang et al., 2007). Jmjd6 catalyzes demethylation of dimethylated arginine residues at histone H3 arginine 2 (H3R2me^2^) and histone H4 arginine 3 (H4R3me^2^). These findings suggested for the first time that arginine residues in histones might be reversibly methylated and demethylated and identified Jmjd6 as the long sought-after eraser enzyme for these regulatory histone marks. All other identified JmjC domain containing histone demethylases target lysine residues in the tails of histone H3 and H4 (H3K4, H3K9, H3K27, H3K36 and H4K20, Greer and Shi, 2012; Kooistra and Helin, 2012) and have been accordingly named lysine demethylases (KDMs) (Allis et al., 2007). The large KDM subfamily of JmjC dioxygenases is grouped based on sequence homology into subfamilies KDM1 to KDM6. The histone lysine residues can be mono-, di-, or trimethylated and are linked to transcriptional activation (H3K4, H3K36, H3K79) or transcriptional inhibition (H3K9 and H3K27) based on their location on promoters and enhancers (for review see, Zhou et al., 2011). Histone arginine residues can be monomethylated or symmetrically or asymmetrically dimethylated depending on the position of one or two methyl groups on the guanidino group of the arginine (**Figure 3**). Asymmetric dimethylation of H3R2me^2a^ associates with transcriptional repression (Guccione et al., 2007; Hyllus et al., 2007) whereas symmetric dimethylation or monomethylation of H3R2 (H3R2me^2s^ and H3R2me^1^, respectively) associates with gene activation (Migliori et al., 2012; Yuan et al., 2012). Asymmetric dimethylation methylation of histone 4 at arginine 3 (H4R3me^2a^) can facilitate p300-mediated acetylation of H3 and H4 at different lysine residues and can thereby promote transcriptional activation (Wang et al., 2001; Li et al., 2010). In contrast, symmetrically dimethylated H4R3me^2s^ strongly inhibits downstream methylations of H3K4 and has thereby been implicated in transcriptional repression (Zhao et al., 2009). Initial evidence for Jmjd6 acting as a histone arginine demethylase (Chang et al., 2007) was based upon incubating recombinant JMJD6 protein with purified bulk histones and synthetic histone tail peptides with various methylated lysine and arginine sites and demonstrated reductions in H3R2me^2^ and H4R3me^2^ methylation levels. These were detected using western blots with histone methylation specific antibodies that did not differentiate between symmetrically and/or asymmetrically dimethylated arginine residues (Chang et al., 2007). A recombinant JMJD6 protein with mutations of the Fe^2+^ binding residues in the JmjC domain did not demethylate H3R2me^2^ and H4R3me^2^ peptides. The results were corroborated using MALDI-TOF mass spectrometry. However, detection of the expected mass shifts on the synthetic H3R2me^2^ and H4R3me^2^ histone peptides required enrichment of the H3R2me^1^ and H4R3me^1^ reaction products with monomethyl-specific antibodies which suggests relatively weak activity. Finally, over-expression of wild-type, but not inactive JMJD6 protein in HeLa cells, produced global reduction of H3R2me^2^ and H4R3me^2^ levels in transfected HeLa cells (Chang et al., 2007). Others have not reproduced Jmjd6 arginine demethylation activity when using H3 and H4 histone peptides in MS-based assays (Webby et al., 2009; Han et al., 2012; Unoki et al., 2013).

A more consistent finding is that JMJD6 can lysyl-hydroxylate histone peptides *in vitro* with no apparent sequence specificity. Comparative amino acid component analysis of purified histones from wild-type and *Jmjd6* knockout mice, revealed differences in monohydroxylation of multiple lysine residues in the tails of histone H3 and H4 (Unoki et al., 2013). This lysyl-5-hydroxylation activity of Jmjd6 on histones was evident in mouse testis, embryos and ES cells suggesting that this new type of histone PTM might be physiologically relevant. 5-hydroxylation of lysyl residues can inhibit subsequent acetylation and methylation at the same site (Unoki et al., 2013) suggesting a role in chromatin configuration and epigenetic regulation of gene expression. Whether Jmjd6 has a role in mediating such histone cross-talk modifications *in vivo* needs to be tested. This will require a genome wide profiling of both active and repressive histone lysine modification marks with ChIP-seq in *Jmjd6* wild-type and deficient cells to determine whether such regulation might occur locally on chromatin. Global assays for alterations of histone lysine methylation marks in *Jmjd6*-overexpressing and *Jmjd6*-deficient cells have so far failed to provide evidence for a function of Jmjd6 as a KDM (Hahn et al., 2010).

**FIGURE 4:**
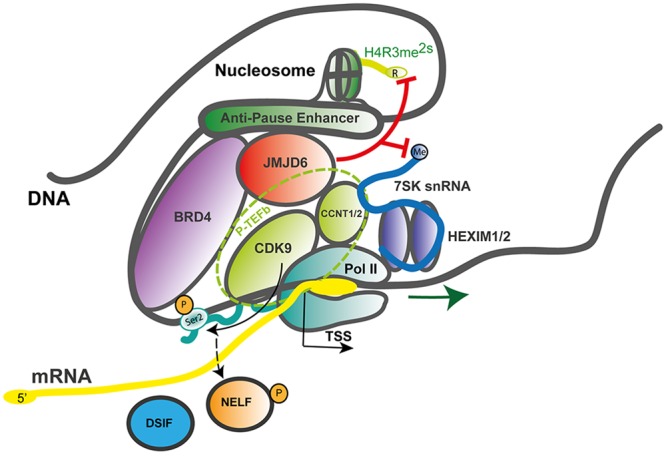
**JMJD6 as a regulator of RNA polymerase II promoter-proximal pause release.** Mechanistic model after Liu et al. (2013) on how JMJD6 regulates transcriptional pause release through dual demethylation activity on H4R3me^2s^ and the 7SK snRNA via interaction with BRD4 on anti-pause enhancers. Demethylation of the 5′-methyl cap of the 7SK snRNA by JMJD6 leads to the dissociation of the positive transcription elongation factor-b (P-TEFb) complex from the HEXIM1/2 polymerase II (Pol II) inhibitory complex. The P-TEFb complex (circled with a dashed green line) consists of the subunit proteins cyclin-dependent kinase 9 (CDK9) and cyclin T1/2 (CCNT1/2). Released P-TEFb phosphorylates serine 2 (Ser2-P) in the C-terminal heptapeptide repeat region of RNA-polymerase II (Pol II) and the negative elongation factor (NELF) which causes its dissociation from the DRB sensitivity-inducing factor (DSIF). Both phosphorylation events facilitate release of paused Pol II and efficient mRNA elongation. In the process of Pol II promoter-proximal pause release recruited JMD6 also demethylates H4R3me^2s^. Genomic DNA with nucleosome and anti-pause enhancer depicted as gray line; mRNA (yellow line); 7SK snRNA (blue line); BRD4, bromodomain-containing protein 4 (purple); DSIF, DRB sensitivity-inducing factor (blue); JMJD6, Jumonji domain containing protein 6 (red); Pol II, RNA-polymerase II (dark green); NELF, negative elongation factor (orange); P-TEFb subunits CDK9, cyclin-dependent kinase 9 (light green) and CCNT1/2, cyclin T1/2 (light green); HEXIM 1/2 dimer, hexamethylene bisacetamide inducible proteins 1 and 2 (violet); Ser2-P, phosphorylated serine 2 of Pol II and TSS, transcription start site; red lines indicating Jmjd6 mediated demethylation reactions.

Jmjd6 might locally demethylate H4R3me^2s^ at certain loci and therefore be associated with transcriptional activation. Jmjd6 appears to act as a regulator of DNA polymerase II (Pol II) promoter-proximal pause release (Liu et al., 2013). Promoter-proximal pausing of Pol II is a sophisticated mechanism in metazoans which regulates the timing and magnitude of transcriptional responses (for review see, Adelman and Lis, 2012; Jonkers and Lis, 2015). After recruitment of Pol II to gene promoters and initial transcription of an mRNA of 20–60 nucleotides in length, Pol II often pauses until additional signals promote productive elongation of the nascent mRNA. Promoter-proximal pause release of Pol II is a major mechanism of transcriptional regulation in response to extracellular stress signals (e.g., heat shock, hypoxia, inflammation) and activation of genes in response to differentiation signals during embryogenesis and development (Liu et al., 2015). In this context, Jmjd6 interacts with the bromodomain-containing protein 4 (Brd4) and components of the positive transcription elongation factor b (P-TEFb) complex, a heterodimer consisting of the cyclin-dependent kinase Cdk9 and a cyclin component (cyclin T1, T2a/b, or K) (Liu et al., 2013). P-TEFb phosphorylates Pol II at serine 2 (Ser2-P) in the C-terminal (CTD) heptapeptide repeat region of the polymerase as well as the negative elongation factor (NELF) which forms a complex with the DRB sensitivity-inducing factor (DSIF) and Pol II, thereby blocking early transcriptional elongation. Brd4 binds to the cyclin T component of P-TEFb and thereby frees P-TEFb from a sequestered inactive ribonucleoprotein complex which contains the small nuclear RNA 7SK (7SK snRNA), the protein HEXIM 1 and the inactive P-TEFb heterodimer (**Figure 4**).

Interactions of Jmjd6 with Brd4 had been reported previously (Webby et al., 2009; Rahman et al., 2011) but the functional consequences of this interaction were unclear. Jmjd6 and Brd4 bind together at distal enhancer regions of a large group of genes called anti-pause enhancers (A-PEs) (Liu et al., 2013). Recruitment of Jmjd6 to A-PEs by Brd4 induced demethylation of the repressive H4R3me^2s^ histone mark. The demethylation activity was also directed at the 7SK snRNA which was shown to serve as a reader of H4R3me^2s^ histone marks. Demethylation of the 5′-methyl cap of the 7SK snRNA was hypothesized to lead to the activation of the P-TEFb complex. Taken together, the data led to a model whereby Jmjd6 demethylation of the 5′-methyl cap induced degradation of the 7SK snRNA leading to dissociation of the 7SK snRNA/HEXIM1 inhibitory complex and retaining of active P-TEFb by Jmjd6 and Brd4 at Pol II pause release promoters (**Figure 4**). Chromosome confirmation capture analysis confirmed a long-range folding of A-PEs to the Pol II paused promoters. Subsequent phosphorylation of Pol II on Ser2-P by released P-TEFb leads to Pol II proximal promoter release and gene activation (Liu et al., 2013, 2015).

Evidence for the catalytic function of Jmjd6 as a H4R3me^2s^ demethylase was provided *in vitro* by incubation of bacterial recombinant or Flag-tagged HEK-293 cell purified Jmjd6 with bulk histones and histone tail derived peptides using histone methylation-specific antibodies and MALDI-TOF MS assays. Importantly, knockdown of JMJD6 in HEK-293 cells produced selective enrichment of H4R3me^2s^ on Brd4 and Jmjd6 co-bound distal enhancers. Jmjd6 mediated demethylation of the 5′-methyl cap of the 7SK snRNA was shown using a dot blot *in vitro* assay with immobilized methylated 7SK snRNA. Knockdown of Jmjd6 or Brd4 *in vivo* increased 7SK snRNA occupancy at distal A-PEs of Jmjd6 and Brd4 specifically at co-regulated genes. The 7SK complex is part of megadalton sized promoter complex which tethers Pol II to genomic DNA near promoter-proximal regions. Serine–arginine (SR) rich proteins have been shown to bind to these complexes and to function in Pol II proximal promoter release. The SR protein SRSF2 (also known as splicing factor SC35) interacts with the 7SK complex at gene promoters and mediates release of P-TEFb in an RNA-dependent manner thereby facilitating Pol II proximal promoter release and transcriptional activation (Ji et al., 2013). In this context, Jmjd6 protein interaction screens identify several SR- and SR-like proteins, including SRSF2/SC35, among others, as binding partners (Webby et al., 2009; Rahman et al., 2011; Heim et al., 2014) (also see Discussion below on the role of Jmjd6 as a splicing regulatory protein). Accordingly, Jmjd6 could have a more general role in the coupling of processes that coordinate mRNA transcription, splicing and 3′-mRNA processing. In a follow up study that extended the discovery of Jmjd6 and Brd4 mediated control of transcriptional Pol II, heat shock protein 70 (HSP70) was found to be required for transcriptional regulation of retinoid acid (RA) induced receptor β2 (RARB2) gene activation by interacting with chromatin and components of the transcription pre-initiation complex (Gao et al., 2015). The monomethylation of a highly conserved arginine residue (R469) in HSP70 was critical for HSP70 mediated recruitment of the transcription factor TFIIH to the pre-initiation complex. Jmjd6 functions as a demethylase of R469me^1^ thus acting in this context as a likely repressor of RA-induced gene activation (Gao et al., 2015). The lack of significant global increases in HSP70 R469me^1^ levels upon knockdown of Jmjd6 in RA-stimulated cells suggests that this demethylation function of Jmjd6 acts again very locally and is context dependent (Gao et al., 2015). Therefore, it might not be a major catalytic activity of Jmjd6 and the regulation of the methylation status of HSP70 and its impact on Pol II transcriptional activity warrants further investigation.

Taken together, functions of Jmjd6 in transcriptional/epigenetic regulation of gene expression have solidified in recent years. However, the question of which are the most biological important functions of Jmjd6 in regulating promoter proximal pause release of Pol II and/or histone modification (arginine demethylation and/or histone hydroxylation) still needs further clarification. This requires careful analysis of Pol II pause release and simultaneous profiling of histone marks in a genome wide context under physiological defined stimuli, preferentially in primary cells and not in immortalized (tumorigenic) cell lines.

## Functions of Jmjd6 in Rna Processing – Regulation of Splicing

**FIGURE 5:**
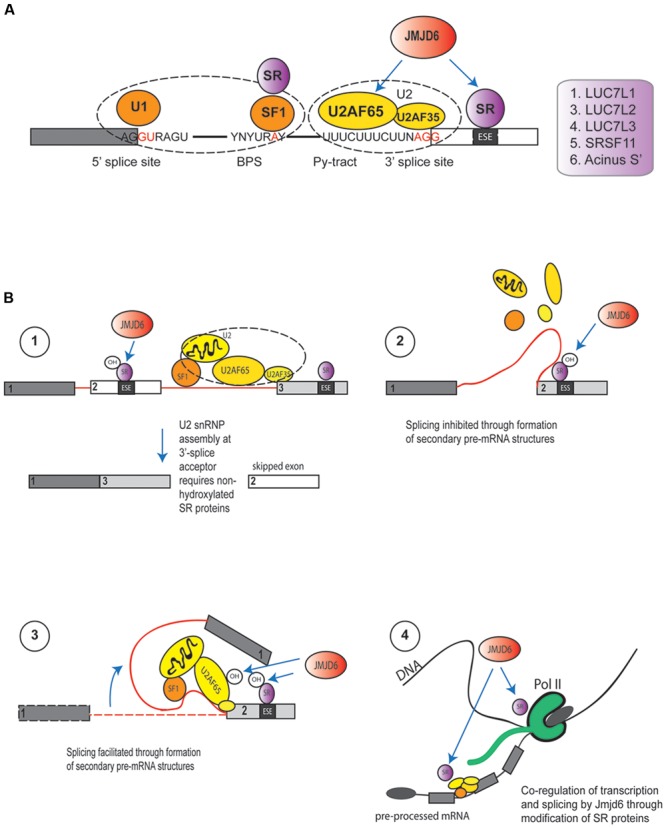
**Functions of Jmjd6 in the regulation of splicing. (A)** Possible role for JMJD6 in modulating binding of splicing regulatory proteins to splice recognition sites on pre-mRNAs. JMJD6 forms a trimeric complex with U2AF65/U2AF35 and has been demonstrated to modulate splicing by lysyl hydroxylation of U2AF65. JMJD6 interacts also with SR proteins that can bind to exon splicing enhancers (ESE) in proximity to 3′-splice acceptor sites. Examples of these SR proteins are LUC7L1, LUC7L2, LUC7L3 (CROP), SRSF11, and Acinus S’ (Heim et al., 2014). JMJD6 mediated hydroxylation of SR proteins might influence selection of exonic 3′-splice acceptor sites through stabilization or weakening of U2AF binding to polypyrimidine tracts. Shown are U1 snRNP binding to exonic 5′-splice donor sites, binding of splicing factor 1 (SF1) and SR protein complexes to intronic branch point sequences (BPS), and assembly of the U2 spliceosome components U2AF65 and U2AF35 together with JMJD6 and SR proteins at the polypyrimidine tract (Py-tract) and 3′-splice acceptor site. Examples of SR proteins that interact with JMJD6 are depicted in the box on the left. **(B)** Hypotheses on mechanisms how JMJD6 could regulate mRNA splicing (see Functions of Jmjd6 in RNA Processing – Regulation of Splicing). Hypothesis (1): Through JMJD6 mediated hydroxylation of SR proteins that bind to exonic splicing enhancers (ESE) exon skipping may be induced. In this case, SR protein hydroxylation by JMJD6 at a local 3′-splice acceptor site might suppress assembly of U2 snRNP components while preferential assembly of unmodified U2 splicing factors and SR proteins might occur at a 3′-splice acceptor site further downstream on the pre-mRNA. Hypothesis (2): Hydroxylation of U2 snRNP components and SR proteins by JMJD6 may induce lopping of pre-mRNAs into different secondary or tertiary RNA structures. JMJD6 mediated hydroxylation of SR proteins bound to exonic splicing silencers (ESS) may facilitate formation of pre-mRNA structures that inhibit splicing of upstream exons. The pre-mRNA folding platform cannot assemble and neighboring exons are not brought into proximity to facilitate splicing. Hypothesis (3): In this case JMJD6 mediated hydroxylation of U2 snRNP and SR proteins triggers the assembly of a platform at exonic splicing enhancers (ESE) that facilitates pre-mRNA looping and recruitment of co-factors needed for exon inclusion into spliced mRNAs. Hypothesis (4): Involvement of JMJD6 in co-transcriptional regulation of mRNA elongation and splicing. Hydroxylation of SR proteins by JMJD6 could regulate polymerase II (Pol II) transcriptional pause release and shuttling of SR proteins from the transcription start complex into spliceosomal complexes where these then regulate splicing reactions.

Proteome interaction screens which have used transient expression of Jmjd6-epitope or Jmjd6-GFP tagged fusion proteins coupled with affinity purification and mass spectrometry (LC–MS/MS) have identified 35 Jmjd6 interacting proteins in at least three out of four independent interaction screens in the human HeLa and HEK-293T cell lines (Heim et al., 2014). The majority of Jmjd6 candidate interactors (63%) were proteins with functional GO annotations in RNA metabolism, RNA processing, and RNA splicing; 16 are arginine-serine-rich (RS) proteins of which nine are known components of the pre-spliceosomal complex A (Heim et al., 2014). Two of the RS-domain containing proteins, U2AF65 and the LUC7-like 2 pre-mRNA splicing factor (Luc7L2) were shown to be lysyl-5-hydroxylated by Jmjd6 (Webby et al., 2009), U2AF65 at lysine residues K15 and K276 and Luc7L2 at K266 and K269 (Webby et al., 2009). The splicing of pre-mRNA is catalyzed by five small nuclear ribonucleoprotein particles (snRNPs U1, U2, U4, U5, and U6) and 100s of accessory proteins that together constitute the spliceosome. The spliceosome complex starts to assemble by recognizing the 5′-exon donor splice site, the intronic branch point and polypyrimidine (PY)-tract and the 3′-exon splice acceptor site (Wahl et al., 2009). In humans, ∼92–94% of protein-coding genes undergo cell or tissue-specific alternative splicing (Wang et al., 2008). *Cis*-acting regulatory sequence elements in the pre-mRNA can have positive or negative effects on the selection of specific splice sites. The SR proteins bind to exonic or intronic splicing enhancers or splicing silencers to stabilize or weaken the spliceosomal complex and control the outcome of splicing (for review see, Fu and Ares, 2014). They possess one or two N-terminal RNA-recognition motifs (RRM) and a C-terminal domain which is enriched in arginine and serine dipeptides (RS domain). Binding of these proteins to the pre-mRNA is mediated by their RRMs while the RS domain is used for the recruitment of other splicing proteins to the spliceosome via protein-protein interactions (Long and Caceres, 2009). Jmjd6 forms a trimeric complex with the U2AF65/U2AF35 heterodimer and interacts with the SR proteins Luc7-like protein 2 (Luc7L2), Luc7L3 (previously known as CROP), SRSF11, and Acinus S’ (Webby et al., 2009; Heim et al., 2014) which are involved in different steps of exon definition and alternative splicing. Jmjd6 binds selectively to the RS domains of these proteins in preference to other SR proteins (Heim et al., 2014). So far, Jmjd6-mediated lysyl hydroxylation of K residues within RS domains has only been shown for U2AF65 (endogenous protein) and Luc7L2 (peptide based) (Webby et al., 2009). There are several ways in which Jmjd6 PTMs might influence splicing (**Figure 5**). U2AF65 is a subunit of the snRNP in the U2 spliceosome that binds to the 3′ splice site of the pre-mRNA (Staknis and Reed, 1994) while LUC7L2 and LUC7L3, similar to most other SR proteins, are regulators involved in AS by modulating the recruitment of snRNPs and by stabilizing the bonding between the U2AF and the polypyrimidine tract (Puig et al., 2007; Bradley et al., 2015). Hydroxylation of these SR proteins by Jmjd6 might control the selection of exonic 3′-splice acceptor sites in the pre-mRNA (**Figure 5A**). SR proteins that are hydroxylated by Jmjd6 at a local exon site on the pre-mRNA might suppress the binding of U2 snRNP components at this position while allowing a preferential interaction of unmodified SR proteins at a downstream splice acceptor site within the spliceosome. The overall net outcome of such a reaction would be the skipping of the upstream exon in the processed mRNA (**Figure 5B**, hypothesis 1). Alternatively, SR proteins that are hydroxylated by Jmjd6 might induce the formation of different pre-mRNA structures. Secondary and tertiary structures of RNA recruit splicing enhancers or silencers to splice recognition sequences which are sometimes 100s or even 1000s of nucleotides apart on the primary transcript (Hiller et al., 2007; Wan et al., 2014). For example, PY-tract-binding protein (PTB) can bind to motifs in RNA and cause looping to repress splicing *in vivo* (Lamichhane et al., 2010). The hydroxylation of U2AF65 and other SR proteins by JMJD6 could possibly provide a platform for pre-mRNA looping and the presentation of different *cis* splice enhancer or silencer elements to the splicing machinery (**Figure 5B**, hypotheses 2 and 3) (Shepard and Hertel, 2008; Fu and Ares, 2014).

In some genes Pol II proximal promoter pause release is associated with immediate downstream assembly of spliceosomal components (David et al., 2011). In these systems, Jmjd6 could regulate recruitment of SR and other components and thereby co-regulate transcription and splicing (**Figure 5B**, hypothesis 4). Future experiments can dissect underlying mechanisms using RNA-protein UV crosslinking and immunoprecipitation (CLIP) approaches coupled with high-throughput sequencing (CLIP-seq, Konig et al., 2011; Cook et al., 2015) and chromatin immunoprecipitation sequencing (ChIP-seq) of selected proteins that constitute the transcriptional machinery. Whatever the precise mechanism, Jmjd6 clearly modulates splicing *in vivo*, mostly through direct interaction with its most validated interaction partner U2AF65. For example, Jmjd6 regulates in an oxygen-dependent manner the splicing of alternative vascular endothelial cell growth factor receptor 1 transcripts (VEGFR1, encoded by *FLT1*) (Boeckel et al., 2011). Reduced Jmjd6 expression, either through knockout, or knockdown in cells, correlated with increased expression of an alternative splice variant of *FLT1* that encodes a protein which lacks the transmembrane and intracellular kinase domain of the normal VEGF-receptor 1 (Boeckel et al., 2011). Jmjd6 was found to mediate *Flt1* splicing through interaction with U2AF65 which was found to bind to *Flt1* mRNA. Even in *Jmjd6*^+/-^ mice, Flt1-dependent angiogenic sprouting was suppressed in a matrigel implant model in comparison to wild-type mice (Boeckel et al., 2011). Jmjd6 appears to act downstream of TNFSF15, a negative regulator vasculogenesis, which promotes expression of the alternative sFlt-1 splice isoform through downregulation of Jmjd6 expression (Qi et al., 2013).

A second example is the regulated splicing of the ferrochelatase gene under iron limiting conditions (Barman-Aksozen et al., 2013). Ferrochelatase catalyzes the terminal step in the heme biosynthesis pathway. It inserts ferrous iron into the heme precursor protoporphyrin IX to form heme B, which is the oxygen carrier component in hemoglobin and an essential co-factor in many proteins and enzymes (Dailey and Meissner, 2013). Partial deficiency in *ferrochelatase* (*FECH*) gene expression results in erythropoietic protophyria (EPP), an inherited metabolic disorder. Under iron deficient conditions aberrant splicing of a mutant *FECH* pre-mRNA variant leads to reduced expression of the FECH protein (Barman-Aksozen et al., 2013). The mutant *FECH* variant has a single nucleotide polymorphism (IVS3-48C/T) immediately upstream of a weak PY-tract in intron 3. The splicing factor U2AF65 binds to a weak PY-tract 5′ to a constitutive splice site and catalyzes the splicing of a predominantly correct transcript (Barman-Aksozen et al., 2013). Under iron limiting conditions, when the JMJD6-mediated U2AF65 lysyl-5-hydroxylation is inhibited, the splice ratio is shifted and a stronger alternative PY-tract is used which leads to inclusion of a partial intron 3-sequence into the *FECH* mRNA (Barman-Aksozen et al., 2013). This transcript variant encodes a premature termination codon (PTC) and is subject to nonsense-mediated decay (NMD). Patients, which carry the intron 3 SNP in *trans* to another deleterious *FECH* allele, are at a particular risk to develop EPP disease. Mechanistically differential binding of U2AF65 to weak or strong PY-tracts is proposed to be regulated by JMJD6. As a Fe^2+^-dependent dioxygenase Jmjd6 is suggested to lysyl hydroxylate U2AF65 at a critical residue (K276) within its second RRM motif which affects the strength of U2AF65 binding to PY-tracts and selection of 3′-splice acceptor sites. Such a regulative function of alternative splicing has been long recognized for a number of other genes (Lewis et al., 2003). In cases where protein expression needs to be regulated tightly, alternative splicing often produces transcripts that differentially undergo NMD. Alternative splice events that target transcripts for NMD to quantitatively regulate gene expression have been termed AS-NMD (alternative-splicing-coupled NMD) or alternatively RUST (regulated unproductive splicing and translation). Interestingly, many splicing regulatory proteins including factors of the core splicing machinery and SR proteins have been reported to autoregulate their own expression through AS-NMD (for review see, Sibley, 2014). This mechanism has been suggested to be important for tissue-specific regulation of splicing patterns and gene expression adaptation to external cues (Boutz et al., 2007). Through its interactions with SR-proteins and their PTM, JMJD6 could have a role in regulating AS-NMD events. The enzymatic activity of Jmjd6 is not only regulated by iron but also by oxygen levels and the availability of its other enzymatic co-factor 2OG (Webby et al., 2009; Boeckel et al., 2011). Moreover, other tricarboxylic acid cycle intermediates such as fumarate and succinate are known to inhibit the enzymatic activity of 2OG dependent dioxygenases (Salminen et al., 2014; Ploumakis and Coleman, 2015). The examples of Jmjd6 regulated splicing of *AIRE, FLT1*, and *FECH*, which all involve mechanisms of intron retention and usage of PTCs leading either to mRNAs subjected to NMD or expression of truncated proteins, suggest that Jmjd6 might have a wider significance in regulating alternative splicing of transcripts that contain PTCs.

## Emerging Roles of Jmjd6 in Cancer

**FIGURE 6:**
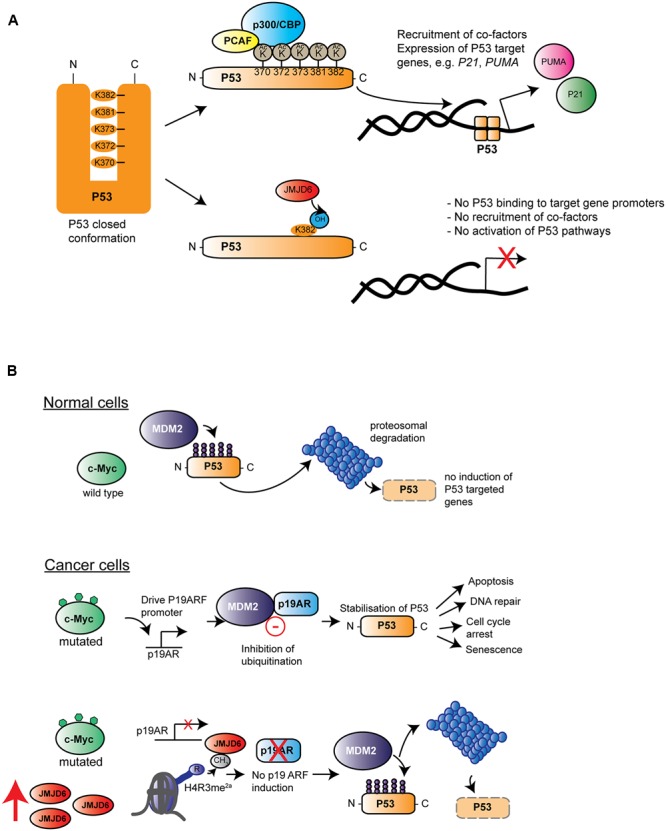
**Roles of Jmjd6 in cancer. (A)** JMJD6 regulates P53 functions by hydroxylation. Inactive P53 is normally locked in a closed confirmation which prevents its binding to target genes on genomic DNA. Acetylation by different acetyltransferases such as p300/CBP (blue) and the p300-CBP associated factor (PCAF, yellow) at C-terminal located lysines (K370-K382) affects P53 transcriptional activity by opening up its closed confirmation exposing its central DNA binding domain. Binding of P53 to gene promoters and recruitment of co-factors induces expression of target genes such as *P21* and *PUMA*. Lysyl hydroxylation of P53^K382^ by JMJD6 has been described to suppress expression of *P21* and *PUMA* by preventing P53 acetylation at this amino acid (Wang et al., 2014). **(B)** Hypothesis of how amplification of Jmjd6 in mouse mammary tumor models inhibits c-Myc induced apoptosis and enhances tumorigenesis. In normal cells (top row) where c-Myc (green) is not mutated, the E3 ubiquitin-protein ligase Mdm2 (Mouse double minute 2 homolog, purple) ubiquitinates p53 thereby leading to its proteosomal degradation. In cancer cells (middle row), upregulated expression of mutated c-Myc induces Cdkna2 (cyclin-dependent kinase inhibitor 2A, also known as p19/14ARF, blue) expression. Cdkna2 binds to Mdm2 and antagonizes its ubiquitin ligase function thereby leading to stabilization of p53. Upregulated p53 induces several important cellular processes linked to tumor suppression (indicated by arrows on the left) foremost induction of apoptosis. In advanced MMTV-Myc induced tumors, the *Jmjd6* gene copy number is amplified leading to elevated levels of Jmjd6 protein (red) expression (bottom row). Through its histone arginine demethylation activity, Jmjd6 demethylates the activating histone mark H4R3me^2a^ at the *Cdkn2a* promoter leading to diminished expression of this Mdm2 inhibitor. P53 is subjected to proteosomal degradation leading consequently to a loss of its tumor suppression function. According to the model of Aprelikova et al. (2016) amplified *Jmjd6* has pro-oncogenic functions through interference with p53 functions and elevated JMJD6 expression in tumor tissue is therefore an unfavorable prognostic marker for cancer prognosis.

Many published studies link high expression of JMJD6 with severe pathological tumor grades, increased tumor growth and/or metastasis (Lee et al., 2012; Zhang et al., 2013; Wang et al., 2014; Poulard et al., 2015; Aprelikova et al., 2016). One candidate mechanism is based upon interaction of JMJD6 and the p53 tumor-suppressor protein pathway (Wang et al., 2014). In colon carcinoma HCT116 cells, p53 was bound by JMJD6 and hydroxylated at lysine 382 (p53^K382^) in its C-terminal domain. The specificity of the JMJD6 catalyzed reaction was demonstrated *in vitro* with recombinant JMJD6 and p53 and *in vivo* on endogenous p53 purified from HCT116 cells by employing LC–MS/MS analyses (Wang et al., 2014). An inactive JMJD6 mutant did not hydroxylate p53 and absence of Fe^2+^ and 2OG from reactions also abolished the p53^K382^ hydroxylation. Hydroxylation of p53^K382^ was shown to antagonize CBP/p300-mediated acetylation at the same residue. Cells depleted of JMJD6 by siRNA showed reduced levels of p53^K382ac^ in immunoblots and reduced binding of p53 to the promoters of its target genes *P21* and *PUMA* (Wang et al., 2014). Acetylation of p53 by CBP/p300 at five lysine residues in its C-terminal regulatory domain has been well-documented to fine tune p53 functions (for review see, Reed and Quelle, 2014). Depending on the particular C-terminal lysine modified, acetylation can modulate the p53 DNA binding affinity and/or co-recruitment of other transcription factors (**Figure 6A**). However, the importance of p53 acetylation on its canonical functions of cell cycle arrest, cell senescence and programmed cell death (apoptosis) are less clear (Reed and Quelle, 2014). HCT116 cells treated with JMJD6 siRNAs were more prone to apoptosis upon challenge with the DNA damaging agent VP16, and showed altered growth in xenotransplantation in nude mice, dependent on the presence of p53. These data together suggest that hydroxylation catalyzed by JMJD6 inhibits the tumor suppressor function of p53 (Wang et al., 2014) (**Figure 6A**).

JMJD6’s contribution to control of apoptosis has also described in mouse mammary tumor models that mimic human breast cancer. A commonly amplified chromosomal region on distal mouse chromosome 11 in tumor samples of three mouse cancer models encompasses the *Jmjd6* gene. This region is syntenic to human chromosome 17q23-qter, which is commonly amplified in human breast cancer patients (Aprelikova et al., 2016). In one model studied in detail, *Jmjd6* was expressed at higher levels in MMTV-Myc transgene derived cell lines compared to controls, probably due to a copy number gain (Aprelikova et al., 2016). c-Myc is a proto-oncogene which can induce cellular proliferation and has pro-apoptotic functions in normal cells experiencing stress (Hoffman and Liebermann, 2008) by inducing expression of the murine p19ARF protein (also known as cyclin-dependent kinase inhibitor 2A; CDKNA2/p14ARF in humans) which binds to the MDM2 E3 ubiquitin ligase that regulates p53 stability (Rizos et al., 2007; Hoffman and Liebermann, 2008) (**Figure 6B**). Jmjd6 overexpression in MMTV-Myc driven tumor cell lines reduces expression of p19ARF, possibly by demethylating an activating H4R3me^2a^ histone mark at the *Cdkna2* promoter leading to reduced p53 expression and increased tumorigenic activity (Aprelikova et al., 2016). This mechanism may or may not also be related to the JMJD6-p53 hydroxylation activity (Wang et al., 2014). Interestingly, Jmjd6 overexpressing tumors also showed reduced amounts of apoptotic cells, thus supporting the argument that Jmjd6 might suppress c-Myc-induced apoptosis. However, at this stage it still needs to be established whether amplification of Jmjd6 and c-Myc driven initiation or progression of tumorigenesis might be a more general mechanism across different types of cancers.

Jmjd6 may also intersect with estrogen receptor signaling in breast cancer, through regulated methylation of the estrogen receptor α (ERα). Methylation of ERα at R260 is critical for the interaction of the receptor with the Src and PI3K kinases and activation of the Akt pathway. Demethylation of ERα by JMJD6 could therefore negatively regulate responses of breast cancer cells to estrogen. Demethylation of ERα by JMJD6 was shown *in vitro* using an anti-ERαR260me^2a^ antibody and recombinant JMJD6 protein (Poulard et al., 2014). A low efficiency of the assay reaction suggested that additional co-factors may be needed that either support the binding of JMJD6 to its substrate or increase its enzymatic activity (Poulard et al., 2016).

Although several studies have suggested a role for Jmjd6 in tumorigenesis, the primary pathways and targets of Jmjd6 that might promote development or progression of cancer are not clear. For example, different studies have reported pro- or anti-proliferative functions of JMJD6 in human MCF-7 breast cancer cell lines (Lee et al., 2012; Poulard et al., 2015). The enzymatic activity of Jmjd6 is likely to be inhibited under oxygen limiting conditions (Webby et al., 2009; Boeckel et al., 2011). This might have implications for the described JMJD6 p53 hydroxylation activity in hypoxic tumor tissue. Several different types of cancer are known to have widespread alterations in alternative splicing either globally or in specific genes. Some of these splicing defects have been linked to occurrence of somatic mutations in genes encoding core spliceosome components or associated splicing factors. Mutations in *U2AF1, U2AF2, SF3B1* and *SRSF2* occur frequently in myelodysplastic syndromes and different types of leukemia (for review see, Dvinge et al., 2016; Scotti and Swanson, 2016). The cBioPortal and COSMIC database^1^ of sequenced cancer genomes shows *JMJD6* mutation frequencies of up to 24% in 62 from 126 of the available Cancer Genome Atlas (TCGA) studies (Cerami et al., 2012; Gao et al., 2013). The highest mutation load (20–24%) is detected in xenograft clones from breast cancer patients and neuroendocrine prostate cancer samples. The majority of detected *JMJD6* alterations are gain-of-copy number mutations (amplifications). Others included deletions and point mutations that are distributed more or less equally over the whole *JMJD6* locus (**Supplementary Figure S2**). There is no mutation enrichment in regions encoding the JMJD6 catalytic center or in particular cancer types with the exception of the aforementioned breast cancer and neuroendocrine prostate cancer samples (**Supplementary Figure S2**). If any of these have functional relevance as true driver events for initiation or progression of cancer needs to be tested. Functional links between Jmjd6 and breast cancer are particularly interesting as deregulation of splicing factors and defects in the crosstalk of the splicing network have been proposed to contribute toward cancer development (Silipo et al., 2015).

## Emerging Functions of Jmjd6 in Infection, Inflammation, and Immune Responses

Studies of Jmjd6 mice indicated a necessary function in thymus and T cell development, probably through the regulation of Aire as discussed above (Kunisaki et al., 2004; Yanagihara et al., 2015). In patients with chronic infections, T cell-mediated immunity can become “exhausted.” In patients with chronic hepatitis B virus infection JMJD6 expression was reduced in T lymphocytes (Chen et al., 2014). Silencing of JMJD6 expression in normal peripheral blood mononuclear cells selectively suppressed the proliferation of CD4^+^ T cells and apparently elevated expression levels of the cycle associated gene, *cyclin-dependent kinase inhibitor 3* (*CDKN3*) (Chen et al., 2014), a known inhibitor of cell cycle progression (Gyuris et al., 1993). The impaired T cell proliferation, and decreased JMJD6 in hepatitis patients was linked in turn, via *in vitro* assays, to elevated circulating platelet-derived growth factor (PDGF-BB) (Chen et al., 2014). However, it is unclear whether the roles of JMJD6 in T cell exhaustion are cell autonomous, and the FANTOM5 data suggests that in humans at least, JMJD6 is much more highly expressed in candidate antigen-presenting cells.

Given its roles in RNA metabolism, and expression in immune cells, Jmjd6 is likely to be required for several aspects of viral RNA replication cycles. Direct evidence of a pro-viral role came from studies of the replication of foot-and-mouth disease virus (FMDV) (Lawrence et al., 2014). FMDV replicates in the cytoplasm and requires host RNA helicase A (DHX9) which interacts with the viral genome and supports its replication (Lawrence and Rieder, 2009). In uninfected cells DHX9 is pre-dominantly located in the nucleus where it is involved in splicing and RNA processing by binding to heterogeneous nuclear ribonucleoproteins. FMDV infection triggers the shuttling of DHX9 from the nucleus to the cytoplasm where it accumulates 3–4 h post-infection (Lawrence and Rieder, 2009). The nuclear-cytoplasmic shuttling of DHX9 is determined by a PTM which involves demethylation of arginine-glycine-glycine (RGG) repeats in its C-terminal nuclear transport domain region (Lawrence and Rieder, 2009). Reciprocal immunoprecipitation confirmed a physical interaction and recombinant JMJD6 was found to demethylate FLAG-tagged and affinity purified DHX9 (Lawrence et al., 2014). Demethylation requires the presence of Fe^2+^ and 2OG, and was inhibited with *N*-oxalylglycine (NOG), a general inhibitor of 2OG-dependent JmjC dioxygenases. Formal proof of the importance of this process still requires analysis of *JMJD6*-deficient cells and cross-validation of the demethylation with LC–MS/MS based analyses on endogenous DHX9 protein (Lawrence et al., 2014).

Within innate immune cells, where Jmjd6 is most highly expressed, one candidate target of arginine demethylase activity is Tumor Necrosis Factor Receptor-associated Factor 6 (TRAF6). TRAF6 is a non-conventional E3 ubiquitin ligase that acts downstream of several important immune pathways including the tumor necrosis factor receptor (TNFR) superfamily, the T cell receptor (TCR), the interleukin 1 receptor (IL1R), and the toll-like receptor (TLR) pathways (Walsh et al., 2015). TRAF6 is reversibly methylated and demethylated at 12 different arginine sites along the protein (Tikhanovich et al., 2015). Methylation of TRAF6 by PRMT1 was shown to inhibit its ubiquitin ligase activity and to suppress the activation of NF-κB target genes. The authors hypothesized that PRMT1 methylation of TRAF6 occurs in naïve cells that are not activated by TLR ligands. This raised the question of whether TRAF6 is demethylated upon TLR activation. Co-immunoprecipitation experiments revealed an interaction between JMJD6 and TRAF6, and overexpression of JMJD6 reduced TRAF6 methylation and ubiquitination in human Huh 7.5 hepatocarcinoma cells which resulted in enhanced NF-κB activation (Tikhanovich et al., 2015). Analysis of TRAF6 methylation kinetics upon stimulation with two different TLR ligands, FSL-1 (TLR6/2 agonist) and lipopolysaccharide (LPS/TLR4 agonist), showed that negative regulation of TRAF6 by arginine methylation is dependent on PRMT1. 30 min after LPS and FSL-1 treatment, PRMT1 protein levels were drastically reduced while JMJD6 expression levels remain constant. Thus, TLR-ligand induced demethylation of TRAF6 seems to be regulated by loss of PRMT1 expression rather than by JMJD6 catalytic activity. Knockdown of JMJD6 decreased NF-κB activation in response to TLR ligands but had no effect on basal pathway activation. This suggested that TRAF6 activity is dependent on the relative abundance of PRMT1 and JMJD6 rather than on the amount or activity of either enzyme alone. The TRAF6 residues R88 and R125 seem to be critical for the negative regulation of TLR signaling by demethylation. However, the question of these residues being direct targets of JMJD6 demethylation still needs to be demonstrated using quantitative LC–MS/MS analyses.

## Concluding Remarks and Future Perspectives

Validating and characterizing Jmjd6 reactions *in vitro* has been a very difficult task. Most analyses have used recombinant or cell purified, tagged Jmjd6 with candidate substrates and its co-factors 2OG and Fe^2+^ in isolation (see **Supplementary Table S1**). In contrast to other members of the JmjC protein family, Jmjd6 has no other accessory domains such as plant homeodomain (PHD) zinc finger domains, AT-rich interacting domains, other types of zinc finger domains or TUDOR domains that control for example substrate specificities of larger histone KDMs (Kooistra and Helin, 2012). It is very likely that Jmjd6 interacting proteins or co-factors are needed to make Jmjd6 reactions *in vitro* more efficient for MS based quantification of reaction products. Here, the whole field has been hampered for more than a decade by the unavailability of specific and well-characterized anti-Jmjd6 antibodies that can be used for co-immunoprecipitation assays or enrichment of Jmjd6 interaction partners and substrates. One way forward is the possibility of insertion of specific small epitope tags into endogenous genomic loci using CRISPR-Cas9-mediated homology-directed repair (Mikuni et al., 2016; Wang and Qi, 2016). A tagged Jmjd6 allele that retains all of its activities and regulation would be a valuable resource. It could be used to experimentally dissect the Jmjd6 substrate repertoire in a given physiological condition or cell type of interest by combining small epitope allele tagging with unbiased proteome assessments *in vivo* (Singh et al., 2015). Making full use of such genetic tools could help to answer questions about the biological importance of multiple Jmjd6-substrate interactions and to gain access to the associated down-stream signaling events and to the mechanisms that control them. Jmjd6 is not unique amongst the JmjC protein family in the diversity of its potential substrates. For example, the ribosomal oxygenases RIOX1 (NO66) and RIOX2 (also known as Mina53) catalyze hydroxylation of histidinyl residues in the 60S ribosomal subunit proteins L8 (Rpl8) and L27a (Rpl27a), respectively (Ge et al., 2012) but also appear to function as histone demethylases for the histone marks H3K4me^1^ and H3K36me^1^ (RIOX1, Sinha et al., 2010) and H3K9me^3^ (RIOX2, Lu et al., 2009). Which are the most important physiological substrates for a 2OG-dependent dioxygenase in a particular condition *in vivo*? Are their functions redundant, or required only to fine-tune other downstream processes such as gene transcription, mRNA splicing, or protein translation? Answers to these questions can only be revealed when appropriate *in vivo* systems for experimental analysis are established which allow the assessment of multiple potential substrates simultaneously. Similarly, the conditional knockout allele of *Jmjd6* will be crucial for the assignment of spatiotemporal Jmjd6 functions and their roles in animal models of disease that cover areas such as cancer, inflammation and infection.

There remain many questions about structure–function relationships for Jmjd6. For example, how does Jmjd6 bind to RNA? Which motifs and regions of the protein are needed for RNA interactions and which different classes of RNA molecules are targets of the protein? Is Jmjd6 regulated by PTMs such as sumoylation and phosphorylation and how does its own auto-hydroxylation activity regulate Jmjd6 oligomerization? The detailed understanding of Jmjd6 substrate interactions requires the structural elucidation of Jmjd6/substrate co-complexes. Such analyses could tell how different substrates can be accommodated in the Jmjd6 catalytic cleft. The catalytic site might be relatively accessible and an extended flexible loop terminal to the first β-strand of its DSBH can be also found in some KDMs providing support for the demethylation activity of the protein (Markolovic et al., 2016).

Recently, a subset of KDMs was discovered, including KDM3A, KDM4E, KDM5C and KDM6B, that can catalyze demethylation of methylated arginines in both histone and non-histone substrates *in vitro* (Walport et al., 2016). This again raises questions about both context specific enzyme activities and functional redundancies *in vivo*. One reason why Jmjd6 is so indiscriminate in targeting so many substrates might lie in its role in carrying out oxygenase functions in large and dynamic ribonucleoprotein (RNP) machineries. Arginine methylation of RNA-binding proteins in RNPs such as transcription initiation complexes, spliceosome and ribosomes is well-established and known to regulate diverse cell and tissue differentiation functions (for review see, Blackwell and Ceman, 2012). Jmjd6, itself an RNA-binding protein, has been established in several independent studies to interact with multiple RNA-binding proteins as summarized above. In some cases, interactions of Jmjd6 with multiple partners in larger complexes might be determined by its interaction with RNA rather than by direct protein-protein contacts. RNase treatment experiments and co-immunoprecipitation studies have shown that the nuclear localization of Jmjd6 and its interaction with particular SR-proteins can be RNA-dependent (Hahn et al., 2008; Heim et al., 2014). Thus, pull-down studies might precipitate larger complexes of Jmjd6 and other proteins that are tightly held together by structural or transcribed RNAs. With the development and further refinement of mass spectrometry methods that reliably use affinity or chemical enrichment of arginine methylated proteins, a more comprehensive and quantitative evaluation of arginine methylomes, in the context of RNA metabolism and RNA processing, might be accomplished (Larsen et al., 2016). Future work in this direction should be guided by genetic analyses which combine *in vivo* studies on biochemical and cellular mechanisms of Jmjd6 in a physiological context.

## Author Contributions

JK drafted the article and prepared the original artwork. AL conceived and wrote the manuscript. MO edited the manuscript and contributed to the critical appraisal of the literature. DH contributed to the interpretation and critical appraisal of database and literature information, contributed text and edited the manuscript. All authors have made substantial intellectual contributions to the concepts presented in the manuscript and have approved the submitted version.

## Conflict of Interest Statement

The authors declare that the research was conducted in the absence of any commercial or financial relationships that could be construed as a potential conflict of interest.

## Footnotes

1 http://www.cbioportal.org/

## Supplementary Material

FIGURE S1 **Tissue expression of human and murine JMJD6/Jmjd6 in BioGPS (biogps.org). (Left)** Expression of *JMJD6* in 79 human tissues based on the Affymetrix GeneAtlas U133A dataset. **(Right)** Expression of murine *Jmjd6* in 61 mouse tissues based on the GeneAtlas MOE430 dataset. Cells and tissues with related ontology are shown in similar colors.

FIGURE S2 ***JMJD6* mutation frequencies as shown in the cBioPortal (http://www.cbioportal.org) of the human Cancer Genome Atlas (TCGA).** The majority of detected *JMJD6* alterations are gain-of-copy number mutations. Green color in columns indicate frequency of SNP mutations, blue color deletions, red color amplifications, and gray color multiple gene alterations. Cancer types from different cancer genome studies are shown at the bottom of the graph.

TABLE S1 **Identified Jmjd6 interaction partners and substrates.** It shows protein and RNA substrates that have been described to be post-translationally modified by Jmjd6. Methods and substrates used in the detection of post-translational modifications (PTMs) are listed. Where endogenous Jmjd6 substrates have been validated, used *in vivo* methods are indicated.
