# Supplementary figures and images for: Jmjd6, a JmjC Dioxygenase with Many Interaction Partners and Pleiotropic Functions
Source: Front Genet. 2017 Mar 16;8:32. doi: 10.3389/fgene.2017.00032 (PMC5352680; doi:10.3389/fgene.2017.00032)

*Homo sapiens*

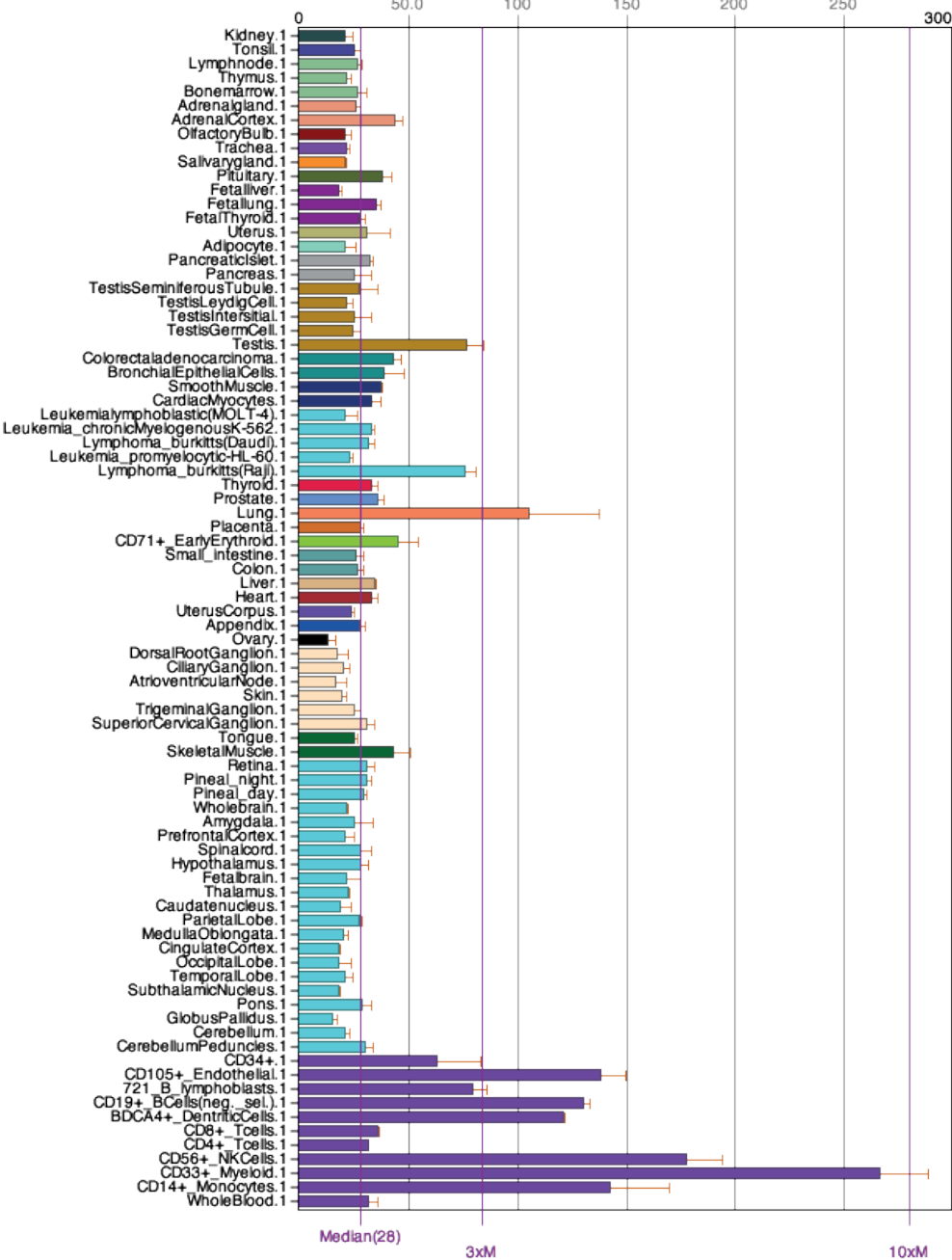

*Mus Musculus*

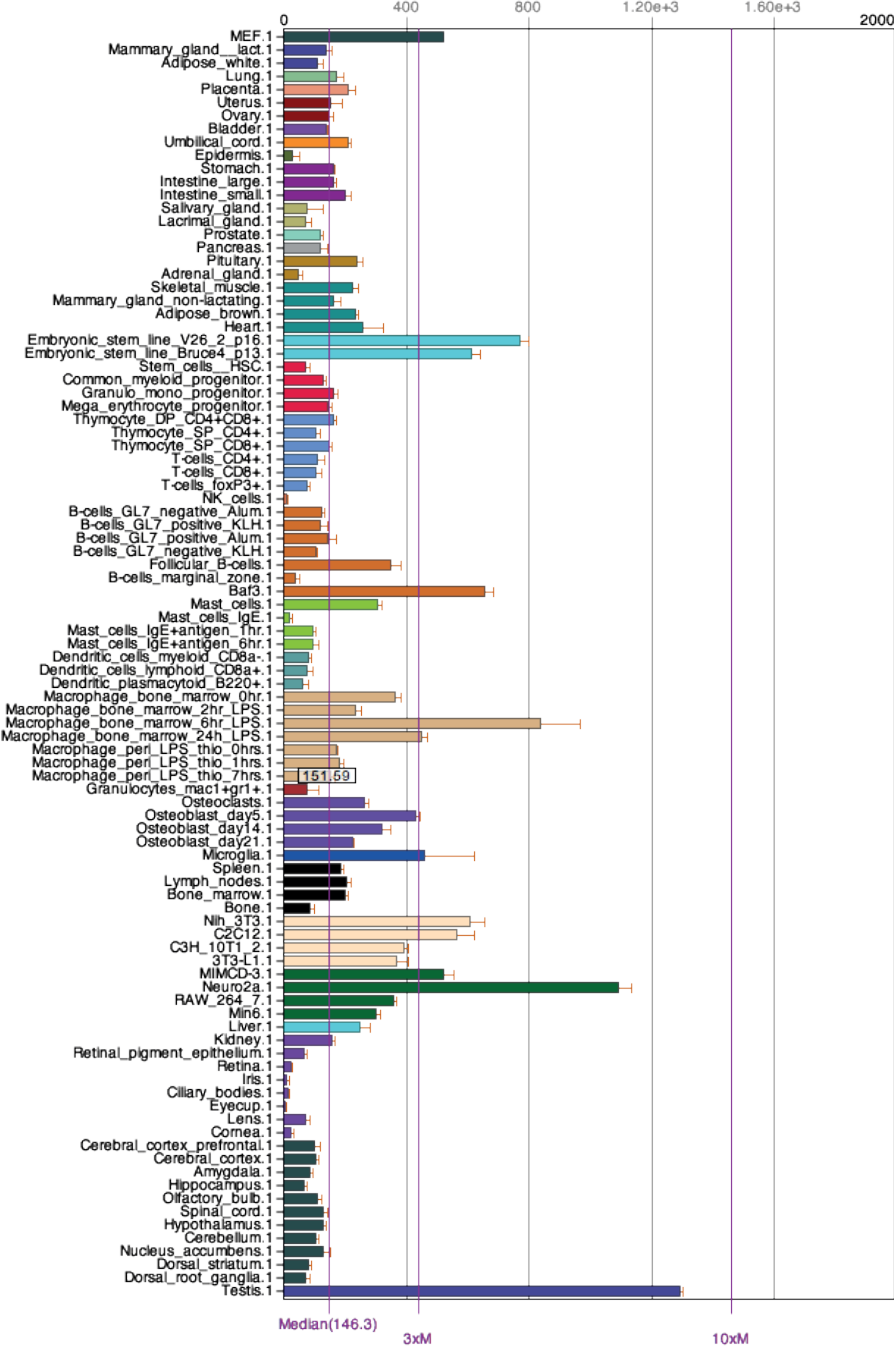

Supplement: FIGURE S1 — Tissue expression of human and murine JMJD6/Jmjd6 in BioGPS (biogps.org). (Left) Expression of JMJD6 in 79 human tissues based on the Affymetrix GeneAtlas U133A dataset. (Right) Expression of murine Jmjd6 in 61 mouse tissues based on the GeneAtlas MOE430 dataset. Cells and tissues with related ontology are shown in similar colors. [file Image_1.PDF]

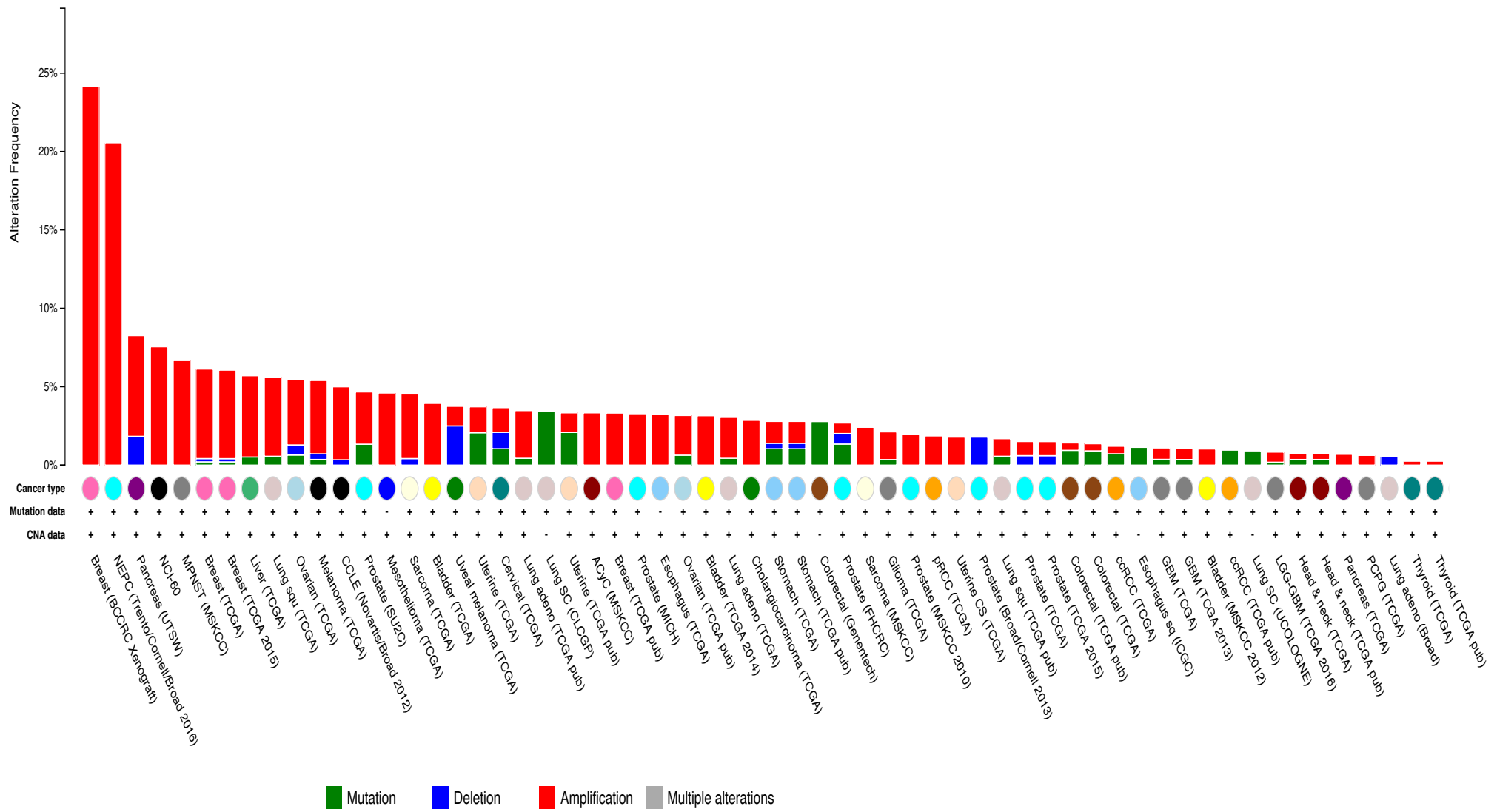

Supplement: FIGURE S2 — JMJD6 mutation frequencies as shown in the cBioPortal (http://www.cbioportal.org) of the human Cancer Genome Atlas (TCGA). The majority of detected JMJD6 alterations are gain-of-copy number mutations. Green color in columns indicate frequency of SNP mutations, blue color deletions, red color amplifications, and gray color multiple gene alterations. Cancer types from different cancer genome studies are shown at the bottom of the graph. [file Image_2.PDF]
