# Supplementary material for: Jmjd6, a JmjC Dioxygenase with Many Interaction Partners and Pleiotropic Functions
Source: Front Genet. 2017 Mar 16;8:32. doi: 10.3389/fgene.2017.00032 (PMC5352680; doi:10.3389/fgene.2017.00032)
Supplement: TABLE S1 — Identified Jmjd6 interaction partners and substrates. It shows protein and RNA substrates that have been described to be post-translationally modified by Jmjd6. Methods and substrates used in the detection of post-translational modifications (PTMs) are listed. Where endogenous Jmjd6 substrates have been validated, used in vivo methods are indicated. [file Table_1.PDF]

Supplementary Table 1: Identified Jmjd6 substrates and described post translational modifications (PTM)

Name	Symbol	PTM catalyzed	Target residue(s)	Assay substrates	Method used	Product IP / antibody*	Reference
7SK small nuclear RNA	7SK snRNA	demethylation	5'-γ-methyl-phosphate cap	*in vitro* transcribed 7SK snRNA	*in vitro*: dot blot hybridization assay	no	Liu et al., 2013
DExH-box helicase 9	DHX9	arginyl demethylation	Multiple arginyl resides in C-terminal DHX9 nuclear transport domain	Flag-tagged purified DHX9 from 293T/17 cells	*in vitro*: immunoblotting with α-DM-RHA antibody which recognizes demethylated Arg residues, Recombinant JMJD6	no	Lawrence et al., 2014
Estrogen receptor 1	ESR1 (ERα)	arginyl demethylation	R260me[2a]	GST-hinge-ERα	*in vitro*: immunoblotting with α-ERαR260me[2] antibody, Recombinant JMJD6	no	Poulard et al., 2014
Histone 2A/2B	n.s.	lysyl-5-hydroxylation	Multiple lysyl residues in histone tail	Cell/tissue purified	*in vivo*: Amino acid composition analysis	no	Unoki et al., 2013
Histone H3	n.s.	arginyl demethylation	H3R2me[2]	Bulk histones Synthetic peptides	*in vitro*: MALDI-TOF MS/MS Recombinant JMJD6	yes α-H3R2me[1]	Chang et al., 2007

Name	Symbol	PTM catalyzed	Target residue(s)	Assay substrates	Method used	Product IP / antibody*	Reference
Histone H3	n.s.	lysyl-5-hydroxylation	Multiple lysyl residues in histone tail	Synthetic peptides<br>Cell/tissue purified	*in vitro*:<br>MALDI-TOF MS/MS<br>Recombinant JMJD6<br>GST-tagged, purified JMJD6<br>*in vivo*:<br>Amino acid composition analysis	no	Unoki et al., 2013
Histone H3	n.s.	lysyl-5-hydroxylation	Multiple lysyl residues in histone tail	Synthetic peptides	*in vitro*:<br>MALDI-TOF MS<br>Recombinant JMJD6	no	Webby et al., 2009
Histone H4	n.s.	arginyl demethylation	$H4R3me^{2}$	Bulk histones<br>Synthetic peptides	*in vitro*:<br>MALDI-TOF MS/MS<br>Recombinant JMJD6	yes<br>α-$H4R3me^{1}$	Chang et al., 2007
Histone H4	n.s.	arginyl demethylation	$H4R3me^{1}$<br>$H4R3me^{2s}$<br>$H4R3me^{2a}$	Bulk histones<br>Synthetic peptides	*in vitro*:<br>MALDI-TOF MS<br>Recombinant JMJD6<br>Flag-tagged, purified JMJD6	no	Liu et al., 2013
Histone H4	n.s.	lysyl-5-hydroxylation	Multiple lysyl residues in histone tail	Recombinant protein<br>Synthetic peptides<br>Cell/tissue purified	*in vitro*:<br>MALDI-TOF MS/MS<br>Recombinant JMJD6<br>GST-tagged, purified JMJD6<br>*in vivo*:<br>Amino acid composition analysis	no	Unoki et al., 2013

Name	Symbol	PTM catalyzed	Target residue(s)	Assay substrates	Method used	Product IP / antibody*	Reference
Histone H4	n.s.	lysyl-5-hydroxylation	Multiple lysyl residues in histone tail	Synthetic peptides	*in vitro*:<br>MALDI-TOF MS<br>Recombinant JMJD6	no	Webby et al., 2009
Histone H4	n.s.	lysyl-5-hydroxylation	H4K8	Synthetic peptide	*in vitro*:<br>MALDI-TOF MS/MS<br>Recombinant JMJD6	no	Han et al., 2012
Heat shock protein 70	HSP70	arginyl demethylation	$R469me^1$	Synthetic peptide	*in vitro*:<br>Immunoblotting with α-$R469me^1$ antibody<br>MALDI-TOF MS<br>Recombinant JMJD6	no	Gao et al., 2015
Jumonji domain containing protein 6	JMJD6	lysyl-5-hydroxylation	K111<br>K167<br><br>K167	Synthetic peptide<br><br><br>Immuno-precipitated JMJD6 from HeLa cells	*in vitro*:<br>MALDI-TOF MS<br>Recombinant JMJD6<br>*in vivo*:<br>LC-MS/MS	no	Webby et al., 2015
LUC7 like 2	LUC7L2	lysyl-5-hydroxylation	K266<br>K269	Synthetic peptide	MALDI-TOF MS<br>Recombinant JMJD6	no	Webby et al., 2009
TNF receptor associated factor 6	TRAF6	arginyl demethylation	Multiple arginyl residues	Immunoprecipitated TRAF6 from JMJD6 overexpressing Huh7.5 cells	*in vivo*:<br>Immunoblotting of immunoprecipitated TRAF6 with pan α-methyl-arginine antibody	yes	Tikhanovich et al., 2015

Name	Symbol	PTM catalyzed	Target residue(s)	Assay substrates	Method used	Product IP / antibody*	Reference
Tumor protein p53	TP53 (P53)	lysyl-5-hydroxylation	K382	Recombinant protein<br>Synthetic peptides<br><br>Immunoprecipitated P53 from HCT116 cells	*in vitro*:<br>LC-MS/MS<br>MALDI-TOF MS<br>Recombinant JMJD6<br><br>*in vivo*:<br>LC-MS/MS	no	Wang et al., 2014
U2 small nuclear RNA auxiliary factor 2, 65 kD subunit	U2AF2 (U2AF65)	lysyl-5-hydroxylation	K15<br>K276	*in vivo*:<br>Immunoprecipitated U2AF65 from HeLa cells	LC-MS/MS	no	Webby et al., 2009

Symbol, n.s. not specified, symbols in brackets give frequently used synonyms
* Antibodies used in immunoprecipitation (IP) enrichment of products from *in vitro* demethylation reactions
